# Transcriptomic response of the wild plant *Mesembryanthemum forsskalii* Hochst. ex Boiss to salinity stress: *de novo* assembly, functional annotation, and transcription factor profiling

**DOI:** 10.1515/biol-2025-1342

**Published:** 2026-06-29

**Authors:** Sumayah I. Alsanie, Faten Dhawi

**Affiliations:** Department of Biology, College of Science, Imam Abdulrahman Bin Faisal University, Dammam, Saudi Arabia; Basic and Applied Scientific Research Center, College of Science, Imam Abdulrahman Bin Faisal University, Dammam, Saudi Arabia; Agricultural Biotechnology Department, College of Agricultural and Food Sciences, King Faisal University, Al-Hofuf, Saudi Arabia

**Keywords:** *de novo* transcriptome assembly, salinity tolerance, *Mesembryanthemum forsskalii*, differentially expressed genes (DEGs), transcription factor (TF) families

## Abstract

Halophytes such as *Mesembryanthemum forsskalii* Hochst. ex Boiss. are well-adapted to harsh environments characterized by high salinity and drought, offering both nutritional and medicinal benefits. However, the genetic and molecular basis of salinity tolerance in *M. forsskalii* remains largely unexplored. This study aimed to generate novel genomic resources and identify the genes, pathways, and transcriptional regulators associated with salt stress tolerance. Plant samples were treated with NaCl at 150 mM and 400 mM, resulting in transcriptomic sequencing yielding over 50 million reads for each concentration. Differential expression gene (DEG) analysis revealed 5,606 and 665 significantly upregulated and downregulated genes, respectively, in plants irrigated with 150 mM NaCl (C vs S1), while 10,730 DEGs were identified under 400 mM NaCl (C vs S2), of which 9,970 were upregulated and 760 downregulated. *De novo* assembly indicated that a substantial proportion of transcripts encoded novel or functionally unknown proteins. Approximately 29 % and 21 % of transcripts under 150 and 400 mM NaCl were functionally annotated in UniProt, GO, and KEGG databases. The most enriched molecular functions included manganese ion binding and oxidoreductase activity. Transcription factor families such as bHLH, NAC and MYB-related family proteins were highly represented. These findings will support salinity research.

## 1 Introduction

The Global Map of Salt-affected Soils shows the location of salt-affected soils (SAS). According to the latest data from 118 nations, that accounts for 85 % of the world’s landmass, approximately 424 million hectares of topsoil and 833 million hectares of subsoil are affected by salt [1]. Inadequate soil management, excessive use of inorganic and organic fertilizers, and irrigation with low-quality water are some of the human activities that lead to soil salinization [2]. “Salinity” is excessive soluble salts in the soil that hampers plant growth [3]. Most terrestrial plants are subjected to salinity stress [4]. Salinity negatively affects plant growth by inhibiting photosynthesis, CO2 absorption, and excessive generation of reactive oxygen species (ROS) [5], [6], [7]. Also, genetic diversity and population clustering are affected by environmental salinity and species-specific genetic adaptations, such as those found in *Tetraena hamiensis* and *T. propinqua* [8]. The harmful consequences of salinity are related to osmotic or water stress, which reduces the capacity of the roots to absorb water. This is followed by ionic toxicity, which results in nutritional imbalance and ROS formation. In addition, salinity causes hormonal imbalances and increases susceptibility to pathogenic infections [9], 10]. Reduced nitrogen (N) intake, NO3− reduction, and NH4+ assimilation as well as a decrease in N metabolism are consequences of salt conditions that impede crop yields [11].

Salinity tolerance in plants is commonly associated with osmotic adjustment, compartmentalization of inorganic ions in vacuoles, accumulation of compatible solutes in the cytoplasm, and regulated transport of Na+ and Cl-through membrane transporters and ion channels [12], [13], [14], [15]. Several signaling pathways are activated in plants in response to salt stress. Such as the salt overly sensitive (SOS) regulatory pathway which regulates ionic homeostasis by modulating Na+/H+ antiporter activity under salt stress [16], 17]. The SOS pathway transports excess Na+ from the cytosol to the apoplast, preventing toxic Na+ accumulation [17], 18]. The SOS signaling pathway includes SOS1, a PM Na+/H+ antiporter, SOS2, a serine/threonine protein kinase, and SOS3 (a calcineurin B-like (CBL) family Ca2+-binding protein) [13], 19]. Crops respond to salt stress via molecular, morphological, and physiological changes [12], [13], [14], [15].

Advancements in next-generation sequencing (NGS) technologies have greatly enhanced our understanding of how plants respond to salt stress. Transcriptome sequencing has been widely used in plant genomic research, and RNA-Seq technology has been used to screen for genes that can withstand high salt levels [20], [21], [22]. For example in *Salsola Drummondii*, the most differentially expressed genes (DEGs) are involved in the breakdown of amino acids within cells or in the response to ROS biosynthesis [23]. In addition, a set of critical genes that may confer salt tolerance by modifying cell wall and root morphogenesis in chickpeas was identified. Moreover, genes that regulate root growth, Ethylene Response Factor (ERF), and PIN-FORMED have been found to engage in phytohormonal crosstalk [24]. Genes involved in plant hormone signal transduction were found in *Solenostemma argel* that had been treated with salt [25]. Furthermore, Toyokura et al. [26] found that 1,100 and 1,394 genes, including CAM pathway, glycolysis, and inositol metabolism, were up- and down-regulated when treated with *Mesembryanthemum crystallinum* for 24-h with 500 mM NaCl. ABA signaling components were also affected by salt treatment. Plant species generally exhibit varying degrees of salinity tolerance, categorizing them into halophytes and glycophytes [27], [28], [29], [30]. Halophytes naturally grow in environments with high salinity levels. And they have adapted special and efficient strategies to cope with salt. It is important to find the specific genes that allow halophytes to tolerate salt and then add them to a related cereal genome to make it more tolerant to salt [31]. RNA sequencing, genome-wide mapping, and advanced bioinformatics programs are some of the modern technologies that have made it easier to decode the plant’s genetic information and come up with possible algorithms to link stress tolerance limit and yield potential.

*Mesembryanthemum forsskalii* Hochst. ex Boiss (locally named Samh), also known as *Mesembryanthemum cryptanthum* [32], 33], belongs to the family *Aizoaceae*, one of the most varied families, containing approximately 2,178 species, and is distributed in numerous regions around the world (GBIF) [32], 34]. *M. forsskalii* is an annual herbaceous halophyte succulent plant [33], 35], predominantly found in arid desert and shrubland habitats [32]. Its seeds have a high nutritional value because they are rich in proteins and carbohydrates [36], 37]. Its flour is used to prepare many traditional dishes in the northern Kingdom of Saudi Arabia [33], 35] and its success in feeding fish [38] and poultry [39] has been demonstrated. Moreover, in diabetic rats, *M. forsskalii* seeds flour diet was hypoglycemic and antihyperlipidemic [40]. additionally, *M. forsskalii* ethanolic seed extract inhibited non-dermatophytes keratinophilic fungi [41].

Considering the nutritional, medicinal, and ecological value of this halophytic plant, as well as its growth in a semi-arid environment where salinity, drought, and heat may co-occur, this study focuses on salinity-responsive transcriptional regulation in this wild species. Although genomic resources for *M. forsskalii* are still limited compared with model plants, salinity-focused transcriptomic information and hub-gene analysis remain scarce. Therefore, this study aimed to generate a *de novo* transcriptome assembly and identify candidate genes, pathways, transcription factor fami-lies, and hub genes associated with NaCl stress responses *in M. forsskalii*. The work is intended to provide a foundation for future validation studies and for exploring stress-tolerance mechanisms relevant to crop improvement and food security.

## 2 Materials and methods

### 2.1 Plant materials and growth conditions

To investigate the transcriptome of *M. forsskalii* Hochst. ex Boiss (Samh) under salinity stress, wild plants were collected from Al-Jouf region in northern Saudi Arabia (29°41′22.0″N 39°35′41.8″E) during the flowering stage (approximately four months old). The identification was carried out by Sumayah I. Alsanie, based on references from both Flora of Saudi Arabia by Migahid [42] and the Flora of Saudi Arabia checklist website [43]. To minimize environmental variability, plants of similar size and growth stage were collected from the same geographical location and carefully transplanted with the surrounding rhizospheric soil into 2-L pots to ensure that experimental conditions closely mimicked the natural environment. The natural collection site is arid to hyper-arid, with low annual rainfall and high evaporative demand; however, soil electrical conductivity and ion composition were not measured at the collection site. This absence of baseline soil salinity data is acknowledged as a limitation when interpreting the relationship between the experimental treatments and natural field salinity exposure. The plant samples were divided into four groups, with three plant-level biological replicates per group before pooling for RNA extraction.

The first group (S1) was irrigated with 150 mM NaCl for 5 days, while the second group (C1), used as the control, was irrigated with distilled water for the same period. The third group (S2) was irrigated with a very high concentration of 400 mM NaCl for 2 days. The shorter exposure period for this treatment was selected to capture early high-salinity transcriptional responses before severe tissue deterioration could compromise RNA quality. The fourth group (C2), serving as the control for S2, was irrigated with distilled water for 2 days. Each plant received 50 mL of either saline solution or distilled water daily, depending on the treatment group. Plants were maintained in a greenhouse at 23 °C under a 12-h light/12-h dark photoperiod. Phenotypic traits such as biomass, tissue ion concentration, and photosynthetic performance were not quantified in this transcriptomic study. Plants were visually inspected at harvest to ensure that leaf tissue remained suitable for RNA extraction, and the lack of quantitative physiological measurements is noted as a limitation. After treatment, harvested tissues were preserved in RNAlater solution prepared from 40 mL of 0.5 M EDTA, 25 mL of 1 M sodium citrate, and 700 g ammonium sulfate dissolved in 935 mL sterile dis-tilled deionized water, adjusted to pH 5.2 using sulfuric acid [44], 45].

### 2.2 RNA extraction and library protocol

For each treatment, equal amounts of vegetative tissue (leaf and stem) from the plant-level biological replicates were combined into one pooled RNA sample before library preparation. Then total RNA was extracted from the tissues using a Qiagen RNA RNeasy kit (Qiagen Inc., USA) according to the manufacturer’s instructions, including RNase-free DNase treatment. The RNA 6000 Nano Kit (Agilent Technologies, Santa Clara, CA, USA) on a 2100 Bioanalyzer (Agilent Technologies) was used to validate total RNA quality with a minimum RIN of 7. RNA concentrations were measured using a NanoDrop ND-8000 spectrophotometer (THERMO Scientific, Wilmington, DE, USA). Total RNA was purified, fragmented, and copied into cDNA (LifeTechnologies, Inc.). The products were purified and enriched with PCR to create the final cDNA library. The tagged cDNA libraries were pooled in equal ratios and used for 2 × 150-bp paired-end sequencing on a single lane of the Il-lumina Novaseq 6000, following the manufacturer’s instructions. Commercial service suppliers (NGB Diagnostics, Noida, India) prepared and sequenced libraries.

### 2.3 *De novo* assembly and mapping

FastQC (version 0.11.5; www.bioinformatics.babraham.ac.uk/projects/fastqc/) was used to check read quality, focusing on parameters such as base quality score distribution, sequence quality score distribution, average base content per read, and GC distribution in the reads. Subsequently, the cleaned reads were subjected to *de novo* assembly using Trinity which is a modular method and software package for *de novo* transcriptome assembly (version 2.11.0; https://github.com/trinityrnaseq/trinityrnaseq/wiki) with default settings of kmer 25. Trinity combines three independent software modules, Inchworm, Chrysalis, and Butterfly, which are sequentially applied to process large volumes of RNA-Seq reads [46]. The assembly was clustered to remove redundancy at a sequence similarity of 90 % using the CD-HIT software (version 4.7, https://www.bioinformatics.org/cd-hit/). The longest isoform was fetched using trinity and was considered a unigene. The unigenes were indexed using BWA (version 0.7.17-r1188; http://bio-bwa.sourceforge.net/). The cleaned data were mapped against the unigenes using the BWA MEM algorithm (https://github.com/lh3/bwa) with default parameters.

### 2.4 Differential analysis and annotation/differential gene expression and annotation of transcriptome

The number of reads mapped to the genes was calculated using SAMtools (version 0.1.19). Differential analysis was performed using DESeq (version 1.30.0), an R package used to analyze count data from high-throughput sequencing assays, such as RNA-Seq, and to test for differential expression. DEGs (Differential Genes) at absolute (log2FC) ≥ 2 and Padjusted value ≤0.05 were considered highly differential significant genes. Annotation was performed using UniProt database (https://www.uniprot.org/). Sequence-based KEGG annotation was performed using the KAAS (https://www.genome.jp/kegg/kaas/) [47].

KEGG and GO enrichment analyses were performed using the R packages clusterProfiler [version 4.6.2; https://bioconductor.org/packages/release/bioc/html/clusterProfiler.html] and topGO [version 2.30.1; https://bioconductor.org/packages/release/bioc/html/topGO.html], respectively.

**Research ethics:** Not applicable.

**Informed consent:** Not applicable.

## 3 Results

### 3.1 Plant growth

**Figure 1:**
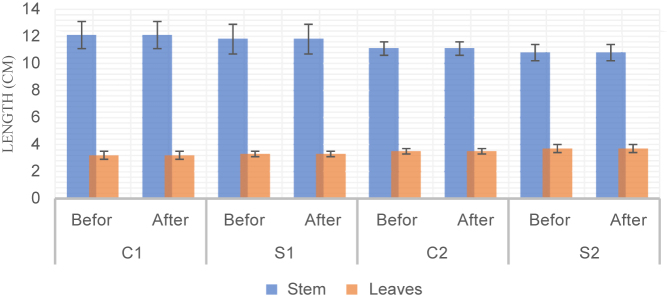
The average stem and leaf lengths of *M. forsskalii* plant samples irrigated with distilled water for 5 days (C1), irrigated with a 150 mM NaCl for 5 days (S1), irrigated with distilled water for 2 days (C2), and irrigated with a 400 mM NaCl for 2 days. The figure shows that no differences in plant growth were observed among the treatments during the treatment period. The error bars show the standard errors.

The average stem and leaf lengths of *M. forsskalii* Hochst. ex Boiss plant samples describe that no differences in plant growth were observed among the treatments during the treatment period (Figure 1).

### 3.2 RNA reads and statistics of sequencing data

Four *M. forsskalii* mRNA libraries representing C1, C2, S1, and S2, were sequenced on the Illumina NovaSeq 6000 platform using 2 × 150-bp paired-end chemistry. The number of raw reads per library ranged from 50,723,132 to 53,055,318. After adapter and quality trimming and removal of reads shorter than 20 bp, 52,029,810, 51,184,011, 50,718,391, and 53,051,369 clean reads were retained in C1, C2, S1, and S2, respectively (Table 1). The GC content ranged from 44 % to 46 %. These sequencing outputs indicate sufficient read depth for *de novo* assembly and exploratory transcriptome profiling; however, the pooled library design limits treatment-level statistical inference.

**Table 1:** Statistics of the transcriptome for the four *M. forsskalii* libraries.

Sample name	Raw reads	Cleaned reads	Read length (bp)	GC% total	Cleaned bases (bp)
C1	52,036,597	52,029,810	20–150	45	15,396,828,243
C2	51,190,275	51,184,011	20–150	44	15,069,268,813
S1	50,723,132	50,718,391	20–150	46	14,997,180,873
S2	53,055,318	53,051,369	20–150	46	15,693,921,564

C1, control, irrigated with distilled water for 5 days; C2, control, irrigated with distilled water for 2 days; S1, irrigated with 150 mM NaCl for 5 days; S2, irrigated with 400 mM NaCl for 2 days.

### 3.3 *De novo* assembly and mapping statistics

The *de novo* assembly of *M. forsskalii* mRNA reads was created using trinity, which produced 429,437 transcripts with an average length of 816 bp and an N50 of 1,606 bp before redundancy reduction. After CD-HIT clustering, 375,104 transcript sequences were retained, with an average length of 723 bp, an N50 of 1,336 bp, a minimum length of 201 bp, and a maximum length of 62,678 bp (Table 2). A total of 295,740 unigenes were identified, with an average length of 558 bp and an N50 of 733 bp. Cleaned reads were mapped back to the unigene set using BWA-MEM, producing mapping percentages of 95.93 %, 95.97 %, 96.15 %, and 96.05 % for C1, C2, S1, and S2, respectively. Although the mapping rates support the internal consistency of the assembly, the high unigene number suggests that the assembly may include fragmented transcripts, isoform redundancy, or lowly annotated sequences, which is typical for *de novo* transcriptome assemblies of non-model plants.

**Table 2:** *De novo* assembly statistics.

Criteria	N50	Total transcripts	Average length (bp)	Min. Length (bp)	Max. Length (bp)
Before clustering	1,606	429,437	816	201	62,678
After clustering	1,336	375,104	723	201	62,678

### 3.4 Differential gene expression and annotation of transcriptome

**Figure 2:**
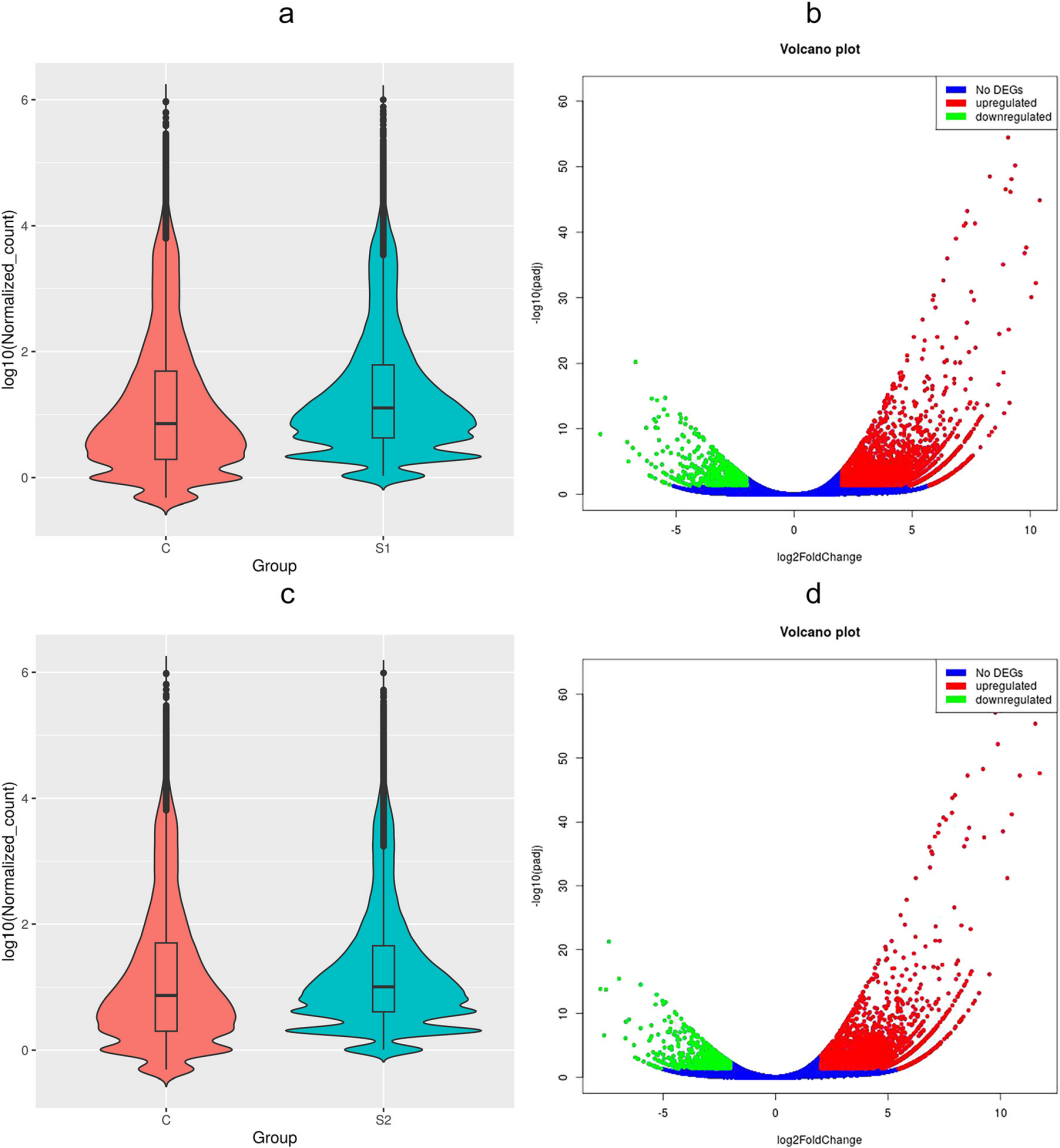
Expression distribution and candidate DEG patterns in *M. forsskalii* under NaCl stress. (a, c) violin plots showing normalized count distributions across pooled control and salt-treated libraries. (b, d) volcano plots showing candidate transcript-level responses to 150 and 400 mM NaCl, respectively. Blue indicates non-DEGs, red indicates upregulated candidate DEGs, and green indicates downregulated candidate DEGs based on |log2FC| ≥ 2 and adjusted *P* ≤ 0.05. Plots were generated using ggplot2 in R.

The R package DESeq (version 1.30.0) was used to identify DEGs under salinity stress conditions compared with control groups combined (C1 and C2 which irrigated with distilled water for 5 days and for 2 days respectively). Volcano and violin plots were constructed using an in-house ggplot R package. The violin plot in Figure 2 shows that the normalized number of genes expressed under the influence of salt (S1 = exposure to 150 mM NaCl, S2 = 400 mM NaCl) was higher than that of the control. and volcano plots showed that most genes were highly expressed compared to the control. Blue, red, and green colors depict non-DEGs (abs (log2FC) < 2 and padj > 0.05), Upregulated Genes at log2FC > 2 with padj ≤ 0.05, and downregulated genes at log2FC ≤-2 with padj ≤ 0.05, respectively.

As shown in Table 3, filtering by |log2FC| ≥ 2 and adjusted *P* ≤ 0.05 reduced the transcript lists to 6,271 candidate DEGs for the C1 versus S1 comparison and 10,730 candidate DEGs for the C2 versus S2 comparison. Under 150 mM NaCl, 5,606 candidate DEGs were upregulated and 665 were downregulated. Under 400 mM NaCl, 9,970 candidate DEGs were upregulated and 760 were downregulated. The larger number of candidate DEGs under 400 mM NaCl is consistent with a broader transcriptional response to severe acute salinity, although the pooled design requires cautious interpretation of DEG counts and adjusted P values.

**Table 3:** Differential analysis statistics in response to *M. forsskalii* under salinity stress.

Comparison	Total DEGs	DEGs (log2FC)	DEGs (log2FC & padj0.05)	Upregulated DEGs [log2FC & padj0.05]	Downregulated DEGs [log2FC & padj0.05]
C vs S1	174,130	91,519	6,271	5,606	665
C vs S2	190,199	106,017	10,730	9,970	760

S1, irrigated with 150 mM NaCl; S2, irrigated with 400 mM NaCl.

Regarding enrichment analysis, 1,803 candidate DEGs in the C1 versus S1 comparison (approximately 29 %) and 2,297 candidate DEGs in the C2 versus S2 comparison (approximately 21 %) were linked to UniProt, GO, and KEGG annotations. The relatively low annotation percentages are consistent with the limited genomic resources available for *M. forsskalii* and suggest that many salt-responsive transcripts may represent uncharacterized, fragmented, or species-specific sequences.

**Figure 3:**
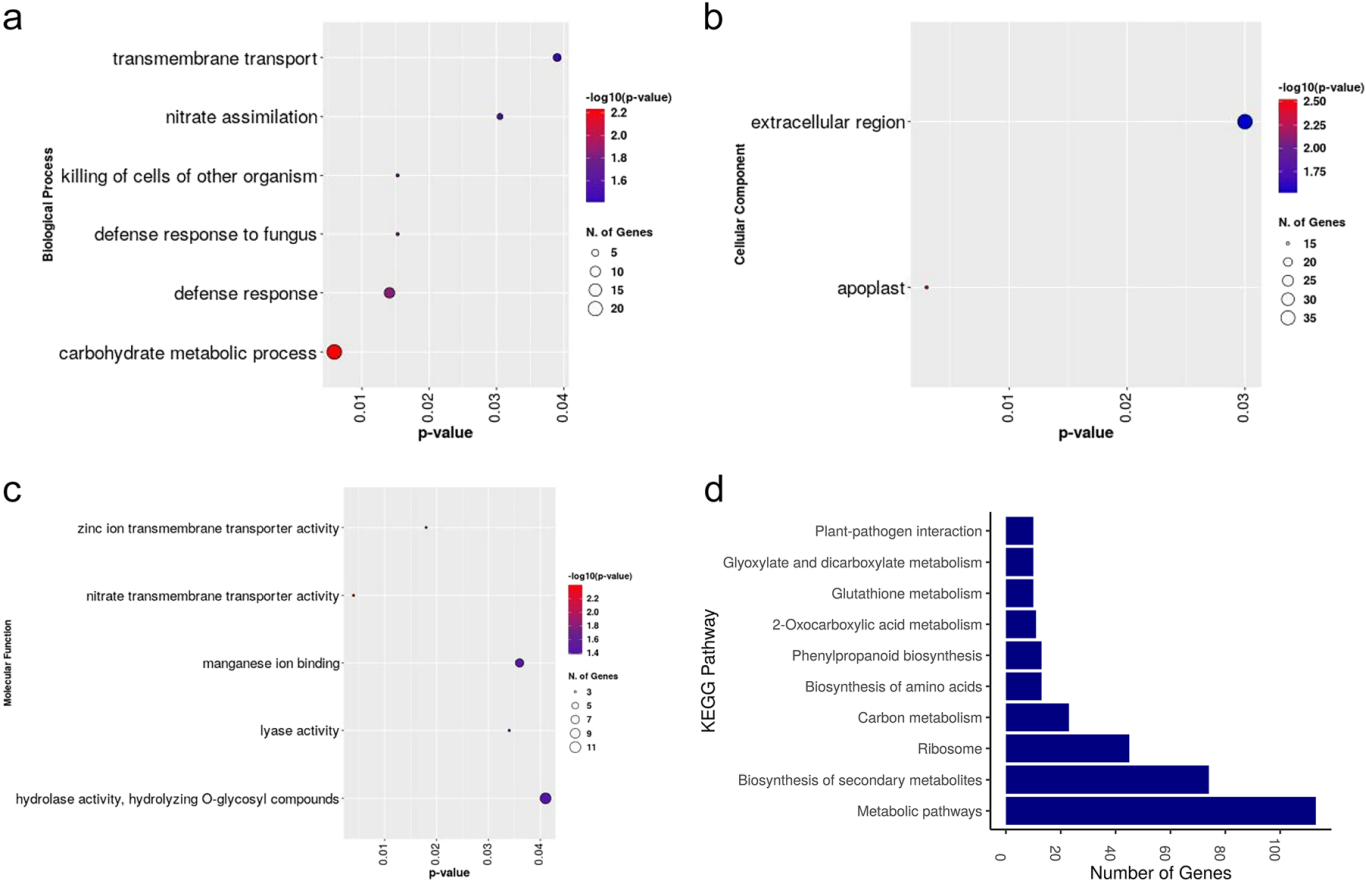
GO and KEGG enrichment analyses for candidate DEGs in *M. forsskalii* exposed to 150 mM NaCl. (a) Biological process enrichment, (b) cellular component enrichment, (c) molecular function enrichment, and (d) top KEGG pathways.

**Figure 4:**
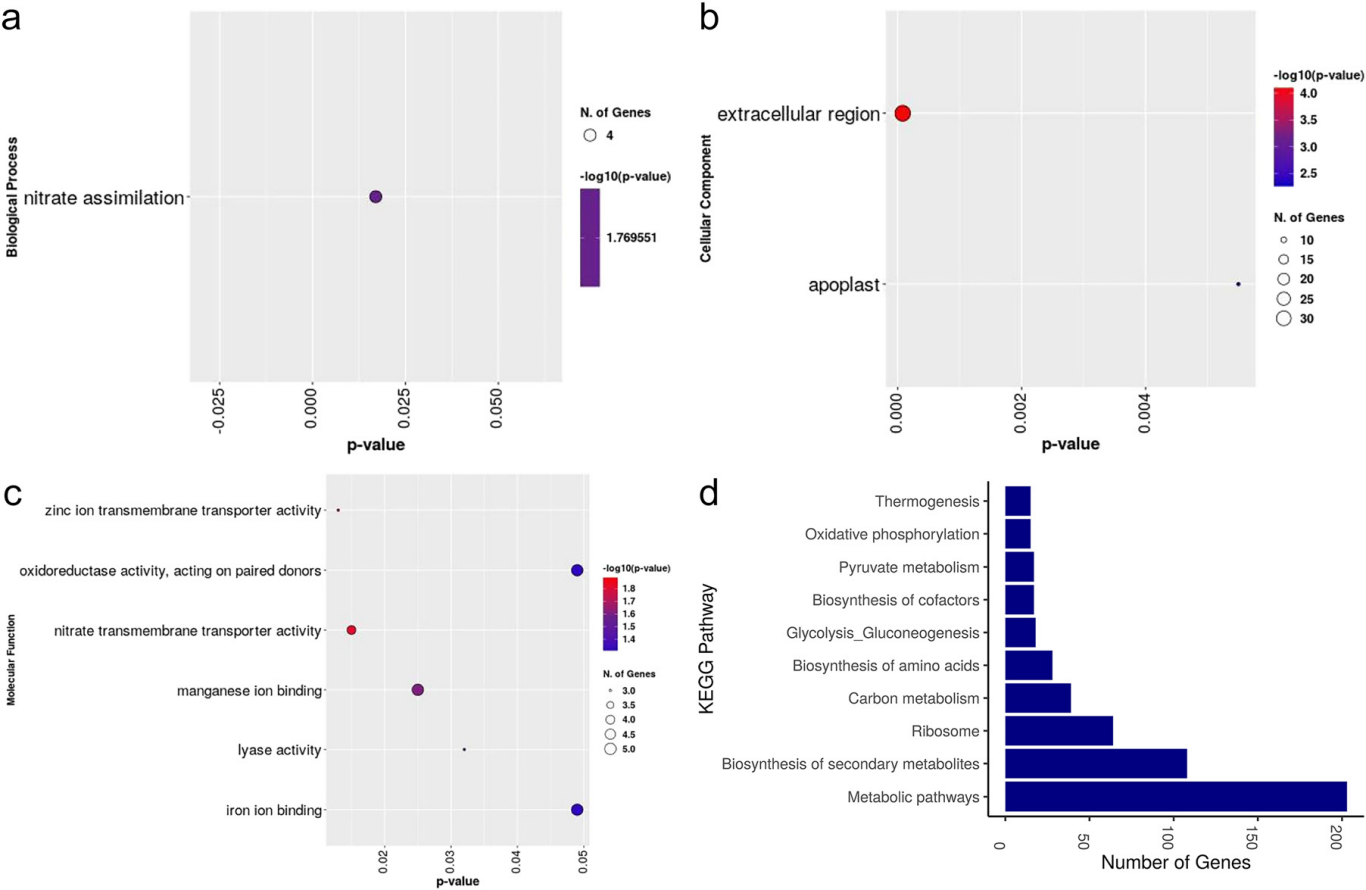
GO and KEGG enrichment analyses for candidate DEGs in *M. forsskalii* exposed to 400 mM NaCl. (a) Biological process enrichment, (b) cellular component enrichment, (c) molecular function enrichment, and (d) top KEGG pathways.

The enriched molecular function (MF) categories included nitrate transmembrane transporter activity, zinc ion transmembrane transporter activity, lyase activity, manganese ion binding, hydrolase activity, and hydrolysis of O-glycosyl compounds. The enriched cellular component (CC) terms included the apoplast and extracellular regions. The top KEGG pathways included metabolic pathways, biosynthesis of secondary metabolites, ribosome, carbon metabolism, and biosynthesis of amino acids. GO categories and KEGG pathways were broadly similar between the two NaCl treatments (Figures 3 and 4). However, nitrate assimilation (GO:0042128) was the most prominent biological process among transcripts responding to 400 mM NaCl, whereas the 150 mM NaCl treatment was associated with several biological processes, including carbohydrate metabolic process, defense response, defense response to fungi, nitrate assimilation, and transmembrane transport. These patterns suggest that moderate salinity may activate a broader suite of defense and metabolic adjustment processes, whereas severe acute salinity may impose a stronger shift toward nitrogen-related and core metabolic regulation.

**Figure 5:**
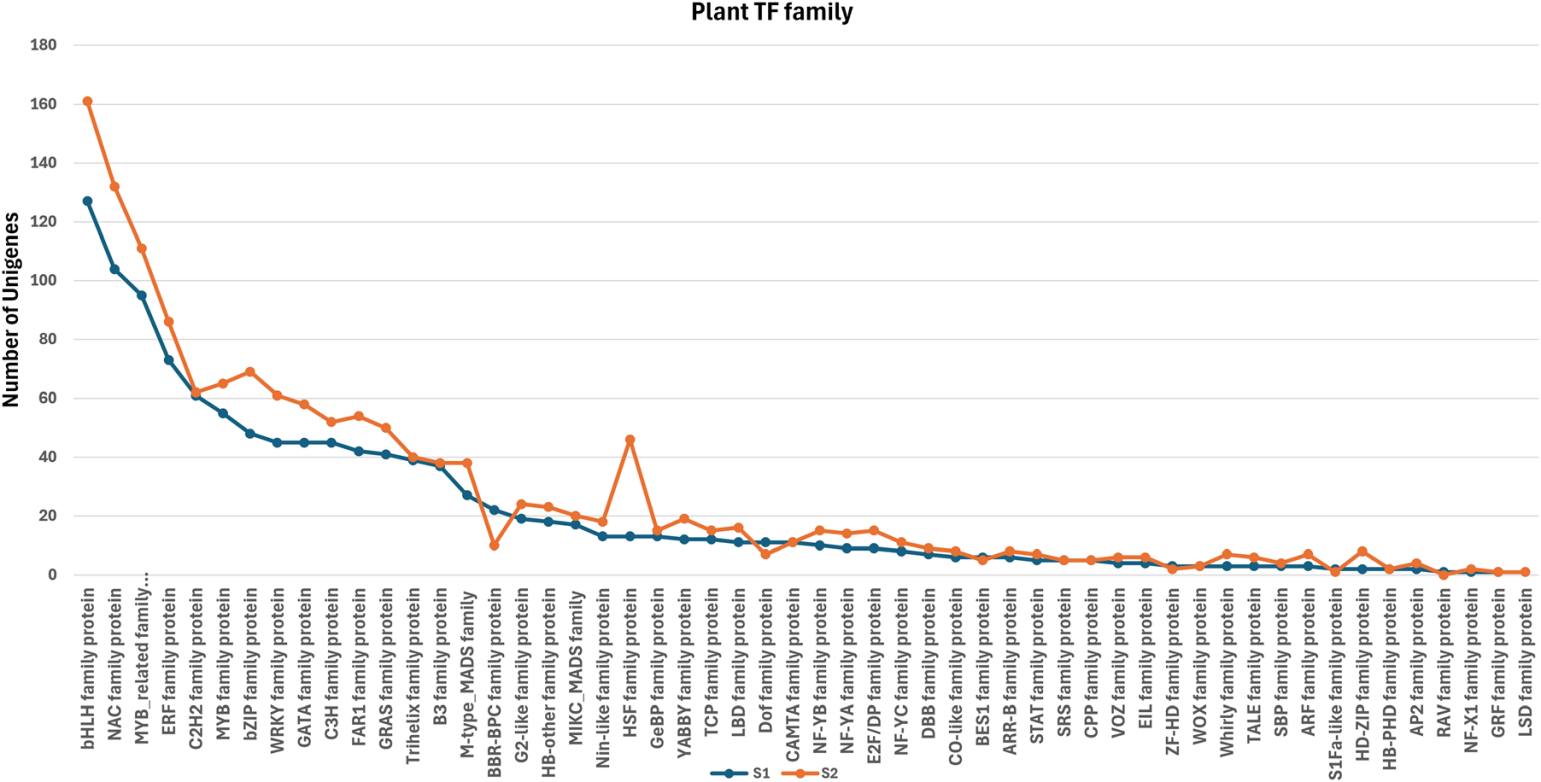
The number of plant transcription factor (TF) families associated with differentially expressed genes (DEGs) when *M. forsskalii* was exposed to 150 mM (S1) and 400 mM (S2) NaCl. The number of unigenes ranged from 161, related to the bHLH family protein, to 1, related to the GRF family protein and the LSD family protein.

The number of plant TF families associated with DEGs varied when *M. forsskalii* was exposed to 150 (S1) or 400 mM (S2) NaCl (Figure 5). In the case of *M. forsskalii* exposed to 150 mM (S1), the number of unigenes ranged from 127 related to bHLH family proteins to one related to GRF family proteins. Similarly, when *M. forsskalii* was exposed to 400 mM NaCl, the highest number (161) was related to the bHLH family proteins, and the lowest number was 1 related to the GRF and LSD family proteins. Most of the top 20 TFs were similar in both treatments (150 and 400 mM NaCl). These included bHLH, NAC, MYB-related, ERF, bZIP, MYB, C2H2, WRKY, GATA, FAR1, C3H, GRAS, Trihelix, M-type MADS, B3, G2-like, HB-other, MIKC_MADS, and YABBY family proteins. The BBR-BPC family of proteins was upregulated (22 unigenes) when *M. forsskalii* was treated with 150 mM NaCl, and downregulated when the plants were treated with 400 mM NaCl. In addition, HSF family proteins were upregulated (46 unigenes) in response to *M. forsskalii* treatment with 400 mM NaCl compared to those treated with 150 mM NaCl (13 unigenes).

## 4 Discussion

### 4.1 Differential gene expression and annotation of transcriptome

In their native environments, halophytes face interacting stresses including salinity, drought, heat, and nutrient limitation. In this study, we exposed *M. forsskalii* to two NaCl treatments, 150 mM for 5 days and 400 mM for 2 days, and used RNA-seq to examine salinity-associated transcriptional responses. Because the species lacks a fully developed reference genome for routine transcriptome-guided analysis, we used *de novo* assembly of cleaned RNA-seq reads. The results showed broad transcriptional reprogramming under salinity, with a larger candidate DEG set under the severe acute 400 mM NaCl treatment. These results are consistent with the expectation that strong osmotic and ionic stress triggers extensive changes in genes related to metabolism, ion transport, ROS detoxification, and stress signaling. However, because the libraries were generated from pooled RNA samples, the DEG results should be interpreted as candidate transcript sets that require independent validation.

This study is considered the first on *M. forsskalii* Hochst. ex Boiss transcriptome analysis; therefore, we used *de novo* assembly using Trinity after RNA sequencing and cleaned the reads. Although no differences in plant growth were observed among the treatments during the treatment period, the results revealed significant difference in gene expression in response to different salinity concentrations compared with the control. This is consistent with Tsukagoshi et al. [48] findings in treating *M. crystallinum* with high salinity concentration. Under salinity stress in *M. forsskalii* (irrigated with 400 mM NaCl), 9,970 genes were upregulated and 760 were downregulated. Although the number of differentially expressed genes (DEGs) was relatively high, the enrichment analysis revealed that only a subset of these genes were significantly associated with key biological processes. Gene Ontology (GO) and KEGG pathway analyses revealed that the upregulated genes predominantly participate in ion transport, several metabolic pathways (including osmoprotectant biosynthesis and cutin, suberin and wax biosynthesis), peroxisome, defense response, antioxidant defense, ATP binding, and transcriptional regulation, all of which are essential for salinity adaptation. On the other hand, the downregulated genes were mostly involved in photosynthesis, several metabolic pathways (including glutathione metabolism and biosynthesis of secondary metabolites), and regulation of flower development, which suggests that there is a trade-off between growth and stress tolerance. These findings highlight that salinity tolerance in *M. forsskalii* involves complex, multi layered transcriptional reprogramming rather than uniform upregulation across the genome. Approximately 480 million RNA-Seq reads from chickpea tissues have been sequenced to identify 3,053 DEGs whose expression changed when plants were stressed with salt. In addition, the significant number of DEGs in the reproductive stage suggests salt-induced transcriptional rearrangement for tolerance [24]. In current study, the common and top molecular function in both treatments was manganese ion binding, and the common and top KEGG pathways were the metabolic pathways. In addition, the top three MF of the DEGs in *M. forsskalii* exposed to 400 mM NaCl were oxidoreductase activity, manganese ion binding, and iron ion binding. These transcriptomic findings support that *M. forsskalii* employs manganese- and iron-dependent mechanisms to cope with salinity-induced oxidative stress, consistent with observations in other plant species. Manganese (Mn) is an essential micronutrient for plants and co-factoring enzymes such as Mn-superoxide dismutase, Mn-catalase, pyruvate carboxylase, and phosphoenolpyruvate carboxylase [49]. Mn improves photosynthesis, antioxidant activity, ion homeostasis, and enzyme activation, reducing salinity [50]. In addition, Eisakhani et al., [51] and El-Beltagi et al., [52] reported that the application of Mn to legume plants increased chlorophyll, protein, and proline levels under salt stress compared with the control. These actions well alleviate the adverse effects. Iron is essential for reducing salinity, drought, and heavy-metal stress. It activates plant enzymatic antioxidants, such as catalase (CAT), peroxidase, and superoxide dismutase (SOD), which scavenge ROS [53].

The biological processes enriched in *M. forsskalii* irrigated with 150 mM NaCl were significantly represented in the GO biological process category, including carbohydrate metabolism, defense response, nitrate assimilation, and transmembrane transport. This is partially consistent with the findings of Sato et al. [54], who identified 18 significantly enriched GO terms in the transcriptome profile of *M. crystallinum* treated with 0.3 % NaCl, mainly related to defense, growth, transcription, post-transcriptional regulation, reproduction, and membrane transport.

In contrast, among the unigenes responding to 400 mM NaCl treatment in *M. forsskalii*, nitrate assimilation emerged as the most significantly enriched biological process in the GO category. Similarly, in *M. crystallinum*, gene expression analyses revealed that salinity enhances the expression of NO_3_^−^ transporters, such as McNRT1 [55].

Overall, the transcriptomic responses of *M. forsskalii* under different salinity levels highlight a complex regulatory network that integrates ion homeostasis, antioxidant defense, and metabolic adjustment. The activation of defense-related and ion-binding pathways, particularly at moderate salinity (150 mM NaCl), suggests a potential molecular basis for stress priming and enhanced tolerance. However, while these findings provide valuable molecular insights, the observed genetic changes may reflect a combination of long-term environmental adaptation mechanisms and acute salt-shock responses. Therefore, further physiological, molecular and agronomic validation is required to determine whether these molecular mechanisms can translate into measurable improvements in plant performance, crop productivity, and stress resilience under field conditions. Accordingly, the present study provides a foundation for future research aimed at linking transcriptomic regulation with practical applications in sustainable agriculture and food security.

The experimental salinity treatments should also be considered in relation to the natural ecology of *M. forsskalii*. This species grows in arid and saline habitats where soil salinity can fluctuate strongly because of evaporation, irregular rainfall, and salt accumulation in the root zone. The 150 mM NaCl treatment was therefore intended to represent sustained high salinity, whereas the 400 mM NaCl treatment was used to capture short-term responses to extreme salt exposure. However, the transcriptomic changes observed here may reflect both halophyte-associated adaptive mechanisms and acute salt-shock responses. Future studies should combine transcriptomics with ion profiling, osmolyte measurements, chlorophyll content, photosynthetic traits, and water-status measurements to better distinguish ecological adaptation from short-term stress responses.

### 4.2 *Mesembryanthemum forsskalii* associated TFs families under salt stress

Regarding the TFs upregulated in response to salt stress in *M. forsskalii* Hochst. ex Boiss, most of the top 20 TFs were similar in both treatments (150 and 400) mMol NaCl). These included bHLH, NAC, MYB-related, ERF, C2H2, MYB, bZIP (top seven), WRKY, GATA, C3H, FAR1, GRAS, M-type MADS, G2-like, HB-other, and HSF. Among these, bHLH, NAC and MYB-related family protein members showed the highest expression fold changes (log_2_FC = 3.6–10.9), suggesting their potential regulatory role in salt stress tolerance. Functional annotation indicated that several NAC, bHLH and MYB-related family protein unigenes were associated with ion binding, ATP binding, GTP binding, RNA binding, and oxidative stress-related pathways, consistent with their established roles in activating downstream stress-responsive genes. Plant TFs, such as bHLH and NAC, which are two of the largest plant-specific transcription factor families, are essential regulators of many biological processes such as abiotic stress [56], 57]. Besides the ERF family, bZIP, MYB, C2H2, WRKY, GATA, FAR1, C3H, HSF, and GRAS family proteins play positive roles in salt stress tolerance in pearl millet [58]. Heat shock factors (HSFs) play a pivotal role in the stress response network in plants [59]. They activate transcriptional pathways associated with salinity tolerance and oxidative defense [60]. The growth state and reactive oxygen scavenging ability of transgenic poplar overexpressing *PtHSF21* were both improved when subjected to salt stress. This enhanced stress tolerance is likely due to the specific binding of HSFs to anti-stress cis-acting elements, known as heat shock elements (HSEs) [61].

Interestingly, members of the BBR-BPC family (22 unigenes) were upregulated under 150 mM NaCl but downregulated under 400 mM NaCl (Figure 5), implying a concentration-dependent regulatory shift. BBR-BPC BPC (Barley B recombinant/Basic Penta cysteine) transcription factors are known to influence organogenesis, cell division, and hormone signaling [62], [63], [64], [65]. The BBR-BPC3 gene family is essential for plant processes such as salt stress response, diurnal rhythm changes, and flowering [62], 66], 67]. And their reduced expression at high salinity may contribute to the inhibition of growth-related processes under severe stress. The observed downregulation of BBR-BPC family members aligns with findings in *Arabidopsis thaliana*, where knockdown of AtBBR-BPC2 improved osmotic stress tolerance [68].

The strong representation of these transcription factor families supports the idea that salt tolerance in *M. forsskalii* is controlled by multiple regulatory layers rather than by a single pathway. NAC, bHLH, MYB-related, ERF, bZIP, WRKY, and HSF transcription factors may regulate downstream genes involved in ion transport, ROS detoxification, osmotic adjustment, protein protection, and stress-related hormone signaling. These transcription factors therefore represent useful candidates for future expression validation and functional analysis. Collectively, these results highlight NAC, bHLH, MYB-related family protein and BBR-BPC families as potential key regulators of salt tolerance in *M. forsskalii*, mediating transcriptional control of ion homeostasis, antioxidant defense, and growth modulation under varying salinity levels.

A key limitation of this study is that plant-level biological replicates were pooled before library preparation, resulting in treatment-level RNA-seq libraries rather than independent biological replicate libraries. This design is useful for generating an initial transcriptomic resource for a non-model halophyte, but it limits dispersion estimation and treatment-level statistical inference. Therefore, the DEG lists, enrichment patterns, and hub genes reported here should be treated as candidate salinity-responsive features. A second limitation is the absence of quantitative physiological measurements, including growth, ion accumulation, osmolytes, and photosynthesis. These limitations have been considered throughout the interpretation and define clear priorities for future replicated validation experiments.

## 5 Conclusions

This study provides the first comprehensive transcriptomic insight into the molecular responses of the wild halophytic plant *M. forsskalii* Hochst. ex Boiss (*M. cryptanthum*) to salinity stress. This study revealed that only 29 % (1,803 genes) of the genes expressed in *M. forsskalii* plants exposed to 150 mM NaCl, and 21 % (2,297 genes) of those exposed to 400 mM NaCl, were successfully identified in the UniProt, GO, and KEGG databases. The top GO categories and KEGG pathways were similar for both treatments, with manganese ion binding and metabolic pathways being the most enriched molecular function and KEGG pathway, respectively. Under 400 mM NaCl exposure, oxidoreductase activity, manganese ion binding, and iron ion binding dominated the molecular functions, while nitrate assimilation represented the most significant biological process. The top 20 transcription factor families were largely shared between both treatments, with bHLH and NAC family proteins being the most represented. Notably, HSF family proteins were upregulated, whereas BBR-BPC family proteins were downregulated under 400 mM NaCl compared with 150 mM NaCl. The enrichment of spliceosome, ribosomal protein, and glutathione metabolism pathways further highlights the central roles of RNA processing, protein biosynthesis, and redox regulation in maintaining cellular stability under salt stress. In contrast, the downregulation of genes associated with circadian rhythm and flowering pathways suggests that salinity may suppress developmental processes to conserve energy for stress adaptation.

Collectively, these findings support the view that *M. forsskalii* Hochst. ex Boiss responds to salinity through coordinated transcriptional, post-transcriptional, metabolic, and redox-related adjustments. However, the conclusions are constrained by the pooled RNA-seq design, the absence of quantitative physiological measurements, and the lack of experimental validation of candidate genes. Future research should validate the identified candidates using qRT-PCR and functional genomics, include independent biological replicate libraries, measure ion accumulation and physiological stress traits, and test responses under combined abiotic stresses such as drought and salinity.
